# Biomaterial scaffolds regulate macrophage activity to accelerate bone regeneration

**DOI:** 10.3389/fbioe.2023.1140393

**Published:** 2023-02-02

**Authors:** Zongtai Liu, Jiabo Zhu, Zhuohan Li, Hanyan Liu, Changfeng Fu

**Affiliations:** ^1^ Department of Spine Surgery, First Hospital of Jilin University, Changchun, China; ^2^ Department of Orthopedics, Affiliated Hospital of Beihua University, Jilin, China; ^3^ Department of Gynecology, Affiliated Hospital of Beihua University, Jilin, China; ^4^ Department of Orthopedics, Baicheng Central Hospital, Baicheng, China

**Keywords:** tissue engineering, bone regeneration, biomaterial, macrophage, bone non-union

## Abstract

Bones are important for maintaining motor function and providing support for internal organs. Bone diseases can impose a heavy burden on individuals and society. Although bone has a certain ability to repair itself, it is often difficult to repair itself alone when faced with critical-sized defects, such as severe trauma, surgery, or tumors. There is still a heavy reliance on metal implants and autologous or allogeneic bone grafts for bone defects that are difficult to self-heal. However, these grafts still have problems that are difficult to circumvent, such as metal implants that may require secondary surgical removal, lack of bone graft donors, and immune rejection. The rapid advance in tissue engineering and a better comprehension of the physiological mechanisms of bone regeneration have led to a new focus on promoting endogenous bone self-regeneration through the use of biomaterials as the medium. Although bone regeneration involves a variety of cells and signaling factors, and these complex signaling pathways and mechanisms of interaction have not been fully understood, macrophages undoubtedly play an essential role in bone regeneration. This review summarizes the design strategies that need to be considered for biomaterials to regulate macrophage function in bone regeneration. Subsequently, this review provides an overview of therapeutic strategies for biomaterials to intervene in all stages of bone regeneration by regulating macrophages.

## 1 Introduction

Bone defects are prevalent clinical manifestations, usually caused by trauma, surgery, and tumor. It can lead to pain, local dysfunction, and even death. Like most tissues in the body, bone tissue has a certain ability to repair and renew itself. Bone can heal well without scarring in the face of some small bone defects, but when the defect area exceeds the critical size or is combined with aging, infection, or metabolic disease, the repair of bone tissue may end up with non-union, mal-union, or delayed union. Previous epidemiological studies suggested the rate of impaired bone healing at 5%–10% (Newman et al., 2021). Although this number may decrease in recent years due to the development of medical technology, it still needs to be taken seriously in low-income countries, elderly, and patients with severe injuries (Ekegren et al., 2018). The financial burden of impaired bone healing is also heavy. In the United Kingdom, the cost of treatment of non-union is approximately £7,000–79,000 per person (Kanakaris and Giannoudis, 2007; Dahabreh et al., 2009; Ekegren et al., 2018). A previous survey in the United States indicated that the treatment cost of tibia shaft fracture patients with non-union was almost twice as much as patients without non-union, and the difference may be even greater when long-term medication and care costs were taken into account (Antonova et al., 2013).

At present, metal implants and autologous or allogeneic bone grafts are still the main clinical methods for the treatment of impaired bone healing. In the United States and Europe, more than half a million patients undergo bone defect repair surgery each year at a total cost of more than US $3 billion (Haugen et al., 2019). In Germany, more than 70,000 autologous or allogeneic bone grafts are performed each year, and autologous bone grafts account for more than 50% (Rupp et al., 2022). Although these treatments appear to be well-established and commercially available today, there are still many problems to be faced. Immune rejection of metallic implants has been effectively controlled in recent years due to continuous improvements in composition, but the different mechanical properties of metallic materials and natural bone tissue tend to lead to stress shielding, resulting in weakening of the surrounding healthy bone tissue (Pacheco, 2019; Liverani et al., 2021). In addition, the secondary removal of metal implants elevates the risk of surgery-related complications, such as anesthesia accidents, infections, and bleeding. Autologous bone graft can well circumvent the rejection problems caused by grafts and has similar mechanical properties and microstructure to normal bone tissue, so it is an excellent material for the treatment of impaired bone healing. Bone donors are primarily derived from bones in non-weight-bearing areas such as the iliac bone, which places a limit on the volume of donors that can be obtained (Schmidt, 2021). In addition, autologous bone grafts may also cause irreparable damage to the donor site. Allogeneic bone grafts can address the need for a large volume of donors to some extent, but this is accompanied by a higher risk of immune rejection and a stable source of donors that is still difficult to address (Penack et al., 2020; Potyondy et al., 2021). With the increasing understanding of bone regeneration mechanisms, the realization of bone regeneration through enhancing the endogenous repair ability of bone tissue has attracted increasing attention (Zhu et al., 2022a; Liu et al., 2022). Macrophage plays a crucial role in the complex regulatory network of bone regeneration composed of a variety of cells and factors, and is a good target for accelerating bone regeneration. The role of macrophage can be simply summarized as “a sweeper, a mediator and a instructor” (Niu et al., 2021).

As a subgroup of immune system cells, macrophages exist widely in various organs of the body, and their important role in tissue repair has been proved by a large number of previous studies (Wynn and Vannella, 2016; Chazaud, 2020). Considering the complex relationship between immune mechanism and bone regeneration, the function of macrophages in bone regeneration is not only manifested in phagocytes (Michalski and McCauley, 2017). Macrophages have a high degree of plasticity and perform different functions under receiving different stimuli (Locati et al., 2020). Macrophages also play a critical role in maintaining the stability of the extracellular matrix (ECM), regulating inflammation levels, immune surveillance, promoting osteoblast proliferation and differentiation, regulating bone formation and resorption, and promoting neovascularization (Lowery and Rosen, 2018; Schlundt et al., 2018; Schlundt et al., 2021). In the complex intercellular and intracellular cascade reactions, the transformation of macrophage phenotype M1/M2 has attracted particular attention. In general, the M1 phenotype showed pro-inflammatory effect and the M2 phenotype showed anti-inflammatory effect. In fact, most of the current studies also focuses on the regulation of macrophage polarization to accelerate bone regeneration. The development of biomaterials provides good tools for interfering with macrophages. In addition, well-designed biomaterials can also provide good local mechanical support, controlled drug release, and bionic cell survival environment (Zhao et al., 2021d). In this review, we summarize the important parameters that need to be kept in mind when designing biomaterials to regulate macrophage activity. Subsequently, we review the therapeutic substance for biomaterial regulation of macrophage and prospected future directions.

## 2 Bone structure and mechanism of bone healing

There are 206 bones in normal adult body, and these bones can be categorized into five categories according to their shape and function: long bone, short bone, flat bone, sesamoid bone, and irregular bone. At the early stage of embryonic development and bone regeneration, woven bone is formed first, which is softer than normal bone and has better elasticity. Later, the woven bone is replaced by mature lamellar bone (Shapiro and Wu, 2019). Lamellar bone is composed of compact bone, cancellous bone, periosteum, and bone marrow. The outer compact bone and the inner cancellous bone are the main components of bone (Wawrzyniak and Balawender, 2022). As the name implies, the compact bone consists of tightly arranged bone plates that are highly resistant to compression and torsion, with Haversian system containing blood vessels and nerves inside. The cancellous bone is composed of honeycomb-like trabeculae with large pores. The arrangement of trabecular bone is closely related to bone stress, which makes the bone achieve good strength with less material (Florencio-Silva et al., 2015; Wawrzyniak and Balawender, 2022). Bone marrow is filled with bone trabeculae and bone marrow cavity of long bones and has hematopoietic function (Pinho and Frenette, 2019; Lucas, 2021). The out surface of bone is covered with dense connective tissue, called periosteum, which has the role of protecting, nourishing, and renewing the bone tissue. Correspondingly, trabeculae and bone marrow cavities are also covered with periosteum, called endosteum, which has osteogenic and osteoclast functions (Wang et al., 2017b; Zhang et al., 2022b).

**FIGURE 1:**
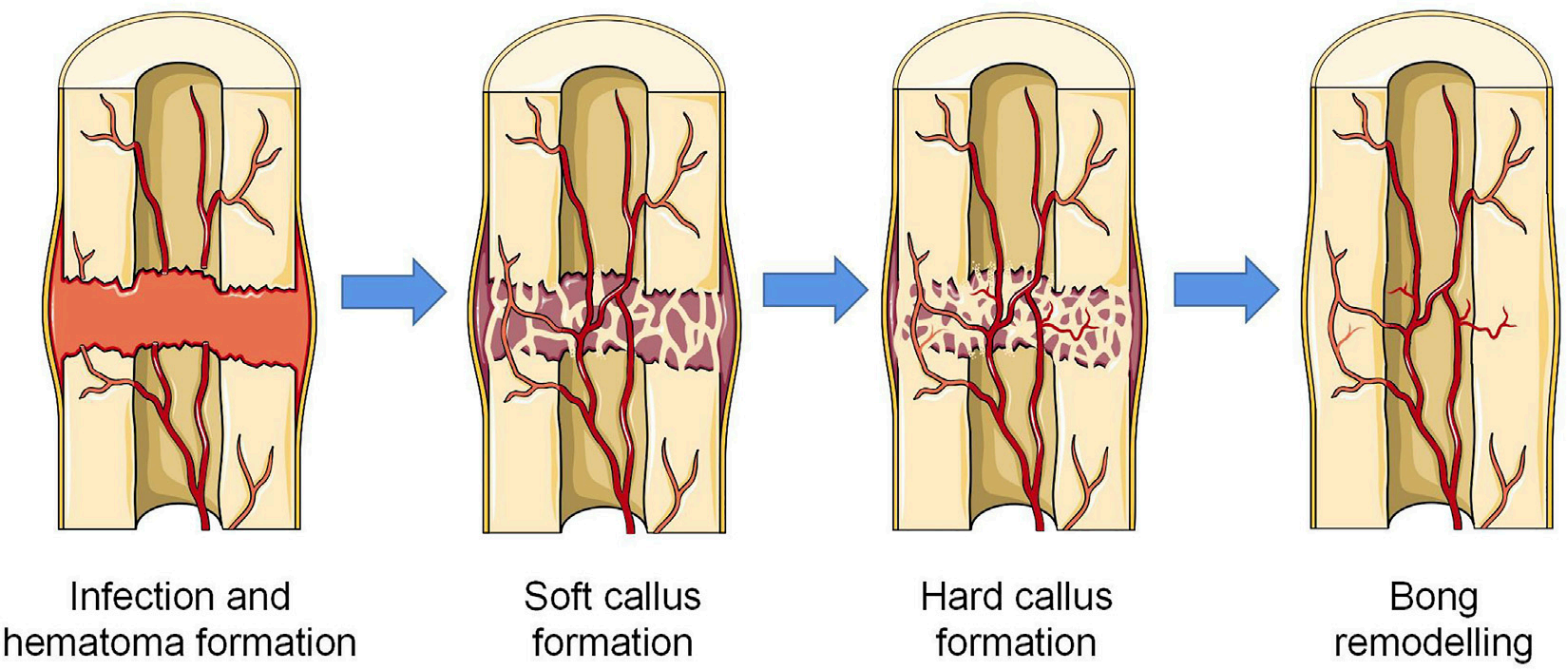
Four stages of endochondral osteogenesis (The Figure was partly generated using Servier Medical Art, provided by Servier, licensed under a Creative Commons Attribution 3.0 unported license).

Depending on the severity of the injury, there are two mechanisms of bone regeneration; intramembranous ossification is seen in the case of minor injury and mechanical stability, and endochondral ossification is seen in the case of severe injury (Salhotra et al., 2020; Marcucio et al., 2023). Intramembranous osteogenesis does not require cartilage as an intermediate product and transitions directly from the initial inflammatory stage to the synthesis and metabolism of bone. Mesenchymal stem cells (MSCs) in the ossification site differentiate into osteoblasts, which produce ECM rich in type I collagen, and with further mineralization of the matrix, the osteoblasts encapsulated in it differentiate into mature osteocytes. At the same time, osteoclasts derived from the hematopoietic monocyte-macrophage system continuously absorb the matrix to ensure the shape and function (Song, 2022). Endochondral ossification requires cartilage tissue as the prerequisite for new bone matrix (Berendsen and Olsen, 2015). The regeneration process can be seperated into four stages: hematoma and inflammation, soft callus formation, hard callus formation, and remodeling (Niu et al., 2021) (Figure 1). In the hours after injury, local bleeding solidifies *in situ* to form a hematoma to prevent further bleeding. In addition, the hematoma provides multiple growth factors to start the subsequent cascade reactions. The necrotic tissue triggers a local inflammatory response in which a variety of inflammatory cells, including macrophages, are recruited, after which the hematoma gradually transformed into granulation tissue (Claes et al., 2012; Newman et al., 2021). Subsequently, chondrogenic cells appear at the fracture site to form hyaline cartilage, and osteoblasts absorb the calcified cartilage to produce new woven bone. Typically, soft callus is formed on the 14th day after the injury. Concurrently, a large number of new blood vessels support the local substance metabolism and the subsequent cascade reaction. As the cartilage is further resorbed and the woven bone is gradually replaced by the lamellar bone, soft callus is gradually replaced by hard callus with higher mechanical strength. In the final bone remodeling stage, with a series of resorption/remodeling events led by osteoclasts and osteoblasts, the structure and function of bone tissue can be precisely regenerated (Einhorn and Gerstenfeld, 2015; Marcucio et al., 2023). The role of macrophages can be found in all stages of bone healing. In the initial stage of inflammation, macrophages are not only responsible for the phagocytosis of tissue debris and foreign pathogens, but also play an important role in promoting angiogenesis and guiding the recruitment and differentiation of MSCs (Niu et al., 2021). In the subsequent callus and remodeling stages, macrophages not only directly differentiate into osteoclasts, but also secrete a variety of factors to regulate the differentiation of osteoblasts and the extracellular matrix microenvironment (Wang et al., 2013; Rucci and Teti, 2016; Xie et al., 2020). Notably, despite the strict chronological order of these phases, there is no separated temporal cut-off point between the phases, and there is a degree of temporal overlap between each phase (Maruyama et al., 2020). This healing characteristic also poses new challenges for promoting bone regeneration.

## 3 Biomaterial design and macrophage regulation

### 3.1 Material design strategy

Although autologous bone grafting is still the gold standard for the treatment of impaired bone healing, the continuous development of tissue engineering can further achieve the regulation of cell activities or local microenvironments based on imitating the structure and function of natural bone tissue (Dimitriou et al., 2011). The use of biomaterials to achieve the multi-functionalization of scaffolds has attracted wide attention in recent years. The materials of scaffolds mainly include metal, ceramic, bio-glass, and polymer (Table.1). In summary, it is difficult to have a single material composition scaffold that can meet the ideal bone regeneration requirements, such as metal materials may lead to stress shielding, difficult degradation of ceramics and bio-glass, and the potential cytotoxicity of polymers (Gracis et al., 2015; Janaszewska et al., 2019; Raffa et al., 2021). Composite scaffolds that combine different kinds of components are expected to solve the performance deficiencies of single materials, while also further increasing the difficulty of scaffold system design and fabrication. For scaffolds to effectively promote bone regeneration by regulating macrophage activity, some properties need to be carefully designed.

**TABLE 1:** Comparison of different implant materials.

Implant material	Major advantages	Major disadvantages
Bone		
Autogenous	Microstructure and mechanical properties of natural bone tissue	The volume of bone obtained in a single surgery is limited. Potential damage to the site that provides the bone Wang and Yeung (2017)
Allogeneic	Adequate bone volume can be obtained. Microstructure and mechanical properties of natural bone tissue	Stable source of bone. Potential immunogenicity and disease transmission Roberts and Rosenbaum (2012)
Metal		
	Good load-bearing capacity and biocompatibility. The source of raw materials is stable and the processing technology is mature	Potential risk of stress shielding. A second surgery may be required to remove it Shafaghi et al. (2019)
Bioceramics/Bioglass		
	Good biocompatibility, chemical stability and wear resistance. The source of raw materials is stable and the processing technology is mature	Refractory to degradation *in vivo*. Poor elasticity and toughness El-Rashidy et al. (2017)
Hydrogel		
Natural composition	Good biocompatibility and degradability. Provide suitable microenvironment for cell survival	Mechanical properties are usually unsatisfactory Yang et al. (2022)
synthetic composition	Many performance parameters can be manipulated manually. Ensure the consistency of different batches of products	Potential cytotoxicity Yue et al. (2020)
Tissue derived scaffold		
	Composition similar to natural bone tissue. Good biocompatibility and low immunogenicity	Limited raw materials, difficult to scale production Jiang et al. (2021b)
Artificial polymer materials		
	Many performance parameters can be manipulated manually. Can be multifunctional. Sufficient supply of raw materials	Complex processing technology and design difficulty. Costly and potentially immunogenic Turnbull et al. (2018)

#### 3.1.1 Parameters to be considered as implants

**FIGURE 2:**
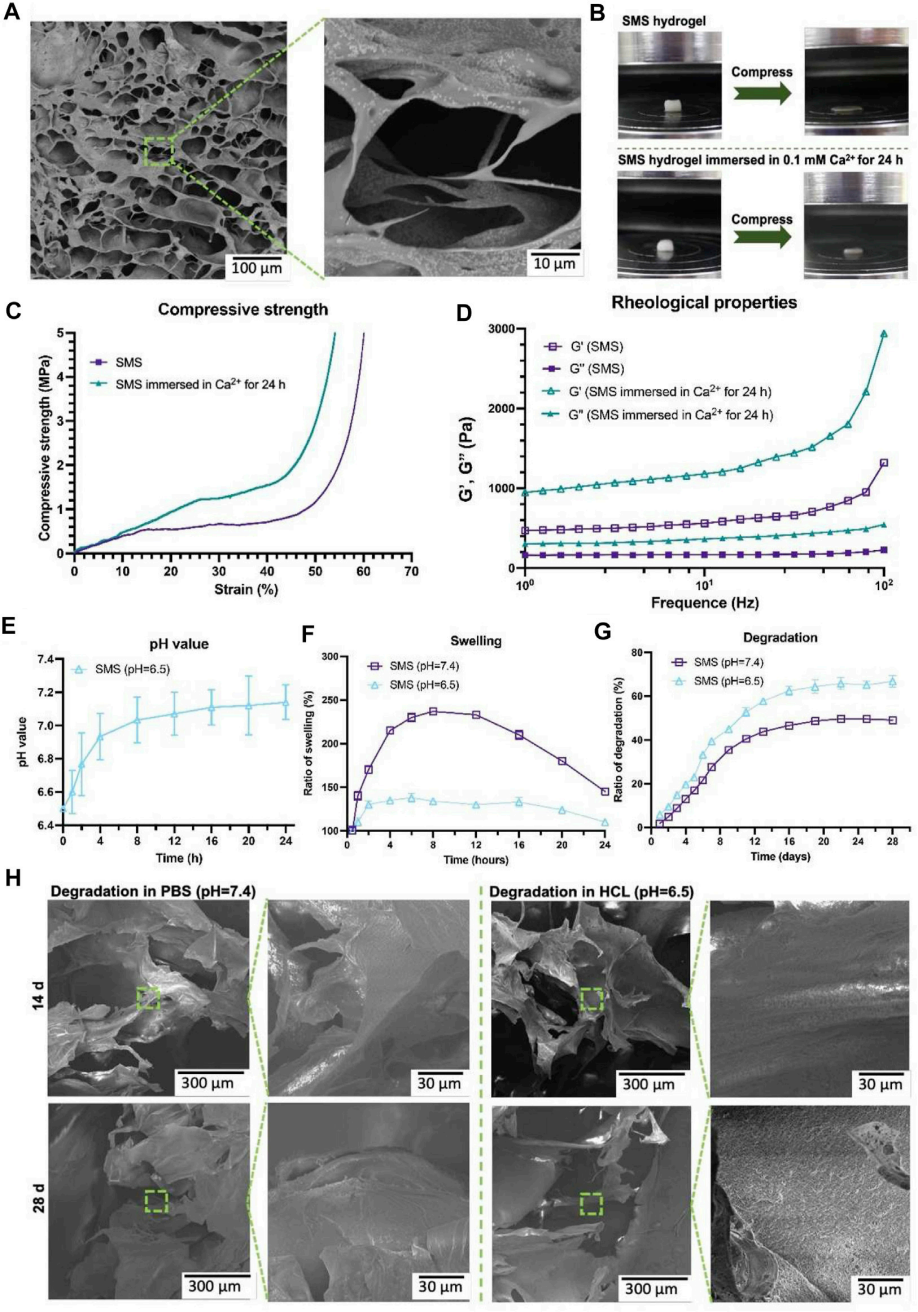
Microstructure of SMS hydrogel under SEM **(A)**. Compression testing diagram **(B)**. Strain curves of SMS hydrogel under different Ca^2+^ conditions **(C)**. Rheological behavior of SMS hydrogel under different Ca^2+^ conditions **(D)**. Effect of SMS hydrogel on PH value in HCl environment **(E)**. Swelling of SMS hydrogel under different PH conditions *in vitro* **(F)**. Degradation of SMS hydrogel under different PH conditions *in vitro* **(G)**. Microstructure of hydrogel under SEM in different PH conditions **(H)**. SMS: silk fibroin/mesoporous bioglass/sodium alginate. Reproduced with permission from (Zheng et al., 2023) (Creative Commons—Attribution-NonCommercial-NoDerivatives 4.0 International—CC BY-NC-ND 4.0).

As biomaterial scaffolds are *in vivo* implants that need to be retained *in vivo* for a period of time, some conventional performance parameters need to be considered first (Table.2). Safety indicators such as biocompatibility, cytotoxicity, and immunogenicity should be the key consideration (Marin et al., 2020; Zhu et al., 2020; Diemer et al., 2021). It is a prerequisite for biomaterials to function without causing local or even systemic adverse reactions *in vivo*. Although many materials have undergone rigorous safety evaluations for clinical use, such as polyethylene glycol (PEG), polycaprolactone (PCL), poly (lactic-co-glycolic acid) (PLGA), and collagen, their safe application in bone defects, not to mention completely new types of composites, still needs to be evaluated in detail, considering the stress, wear and tear and the local inflammatory microenvironment reacts with the biomaterial (Ho-Shui-Ling et al., 2018; Zhao et al., 2021b). Ideally, the scaffold *in vivo* will be completely replaced by new bone tissue to return to normal anatomical structure and function, so the biomaterial scaffold should be biodegradable. It is worth noting that the degradation rate should be in line with the rate of bone regeneration, too fast degradation will be difficult to provide effective support, and too slow degradation will impede the growth of blood vessels and bone matrix. Pre-adjusting the composition or physical parameters of composite materials or correlating the decomposition reaction with the substance which changes significantly in bone regeneration are common approaches to regulate the decomposition rate of materials. Miao et al. (2021) successfully controlled the time scale of scaffold degradation *in vivo* by adjusting the porosity and phase composition of strontium-doped tricalcium phosphate (Sr-TCP) microspheres in a composite scaffold system. Zheng et al. (2023) designed a composite hydrogel system to achieve adaptive reinforcement and degradation by responding to Ca^2+^ concentration and pH in the inflammatory microenvironment (Figure 2). Infection is an important cause of impaired bone healing. Serious contamination of open wounds, improper nursing of surgical incisions after operation, and low immunity of patients may lead to infection. Biomaterial with antibacterial activity is not a new concept. The concept of biomaterials with antimicrobial effects is not new and can be achieved by various means such as biomaterials combined with antibiotics, antimicrobial peptides, and metal particles with antimicrobial effects. Wu et al. (2021) achieved long-lasting antibacterial effects by loading N-halogen polymer coatings on the surface of titanium implants. Similarly, Hayashi et al. (2022) modified the surface of carbonate apatite with silver phosphate to make the scaffold exhibit good antibacterial activity. Interestingly, they also reported that the honeycomb structure also had the effect of preventing bacterial growth. The biomaterial should also have a certain mechanical strength, considering that the graft may have weight-bearing or support requirements *in vivo*. The biomaterial should mimic the mechanical properties of natural bone tissue as much as possible. The utility of biomaterial scaffolds in bone regeneration is usually evaluated from three dimensions: osteoinduction, osteoconduction, and osseointegration. These dimensions range from the induction of MSC differentiation at the cellular level to the binding of the graft to the local tissue of the host at the tissue level. It is worth emphasizing again that no material can satisfy all desirable design parameters. In practical clinical applications, it is often necessary to seek a balance between various parameters according to actual needs.

**TABLE 2:** Parameters affecting the regulation of macrophage function by biomaterials.

Parameter	Function
Parameters to be considered as implants	
Biocompatibility	Ensure the safety of the implant Marin et al. (2020)
Cytotoxicity	Ensure the safety of the implant Diemer et al. (2021)
Immunogenicity	Ensure the safety of the implant Gao et al. (2021)
Biodegradablity	Provide proper support and space for new tissue Miao et al. (2021)
Antibacterial property	Ensure the safety of long-term implantation and assist in the treatment of possible co-infection of bone defects Wu et al. (2021)
Parameters to regulate macrophage	
Degradation product	Influence macrophage phenotype and secretion Chen et al. (2014)
Pore size	Influence macrophage morphology, infiltration and polarization Tylek et al. (2020); Li et al. (2022)
Substrate stiffness	Influence macrophage migration, polarization and phagocytosis Sridharan et al. (2019)
Surface roughness	Influence macrophage attachment and polarization Wang et al. (2018)
Surface wettability	Influence macrophage phenotype and secretion Lv et al. (2018)
Material topography	Inflience macrophage attachment, morphology, polarization and secretion Chen et al. (2010)
3D structure	Influence macrophage morphology, fusion, infiltration and polarization Jiang et al. (2016). Provide a microenvironment close to the body
Material group charge	Influence macrophage polarization and osteogenic differentiation Mao et al. (2022)
Material group chirality	Influence macrophage phenotype and secretion Jiang et al. (2022)
Physical stimulation	Influence macrophage morphology, migration, polarization and secretion Hoare et al. (2016); Wosik et al. (2018)

#### 3.1.2 Parameters to regulate macrophage

**FIGURE 3:**
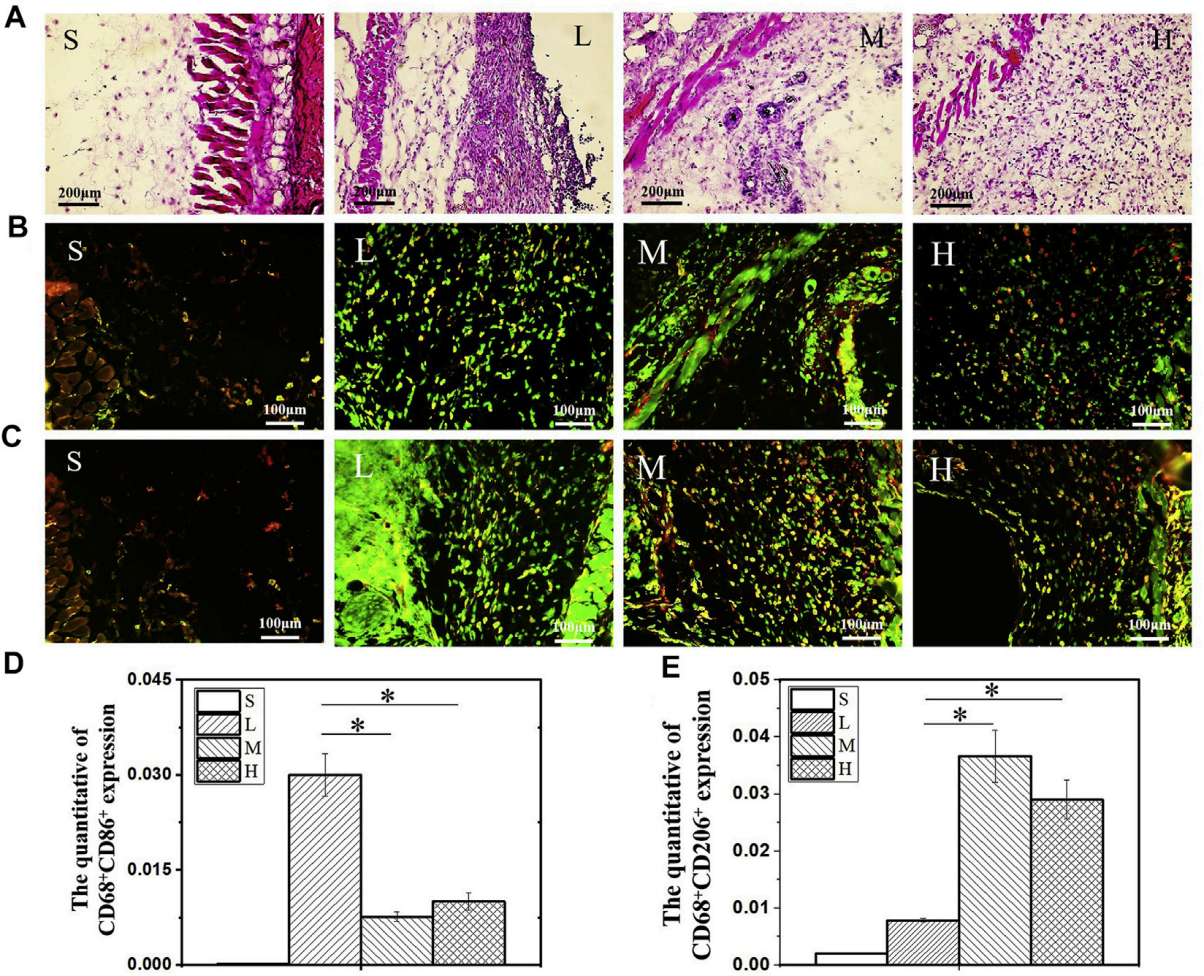
H & E staining image after 14 days of hydrogel subcutaneous implantation **(A)**. Immunofluorescence staining image of CD68^+^CD86^+^ **(B)** and CD68^+^CD206^+^ **(C)** macrophages after 14 days of hydrogel subcutaneous implantation. Quantification analysis of CD68^+^CD86^+^ **(D)** and CD68^+^CD206^+^ **(E)** macrophages. S: sham, L: low stiffness, M: middle stiffness, H: high stiffness, *n* = 3, *p < 0.05. Reproduced with permission from (Chen et al., 2020).

As scientists increasingly focus on the influence of material properties on macrophage activity, exploring the corresponding parameters adapted to the macrophages and applying them will undoubtedly lead to better regulation of macrophage activity (Table.2). Substances produced during scaffold degradation may be absorbed by macrophages or affect the local microenvironment. Meanwhile, scaffolds that are difficult to degrade often cause local chronic inflammation, which is an important adverse effect on bone regeneration. Wang et al. (2017a) reported that bidirectional calcium phosphate (BCP) ceramics promoted migration and osteogenic differentiation of MSCs by promoting the secretion of macrophages. Chen et al. (2014) demonstrated that Ca^2+^ generated from β-tricalcium phosphate (β-TCP) degradation can promote macrophage switching to the M2 phenotype and increase bone morphogenetic protein (BMP)-2 expression by activating calcium-sensing receptor (CaSR). Similarly, the effects of Mg^2+^, Zn^2+^, Sr^2+^, and Cu^2+^ on macrophage phenotype and secretory function have been demonstrated (Huang et al., 2019; Bai et al., 2021; Tan et al., 2021; You et al., 2022). To provide living space for new blood vessels and tissues, scaffolds often have certain pores, and the size of the pores will have an impact on macrophage activity. However, there are different conclusions about the suitable pore size for macrophages. Tylek et al. (2020) reported that scaffolds promoted differentiation of M2 phenotype macrophages when the pore size was reduced from 100 to 40 μm. Similarly, Wang et al. (2014) reported that 30 μm pore size was beneficial to the phenotypic differentiation of M2 macrophages. However, in the study by Li and co-workers (Li et al., 2022), scaffolds with the pore size of 600 μm had better M2 cell infiltration. These differences may be due to the different raw materials and fabrication processes of the scaffolds. The substrate stiffness provided by the scaffold also affects the activity of macrophages. Sridharan and co-workers (Sridharan et al., 2019) explored the effect of material stiffness on macrophage polarization, migration pattern, and function. The results indicated that macrophages cultured on high stiffness substrates (323 kPa) exhibited slow mesenchymal migration with pro-inflammatory phenotype and impaired phagocytosis, while macrophages cultured on softer substrates (11 kPa and 88 kPa) exhibited fast amoebae migration with anti-inflammatory and highly phagocytic phenotype. Considering that macrophages may adapt to different stiffness in different tissues. (Chen et al. (2020) compared the effects of different stiffness substrates on bone marrow-derived macrophage function. At low stiffness (2.55 ± 0.32 kPa), macrophages were more likely to differentiate into M1 phenotypes and secrete more pro-inflammatory factors, while at medium stiffness (34.88 ± 4.22 kPa), macrophages showed more M2 phenotypes and secrete more anti-inflammatory factors (Figure 3). Zhao et al. (2021a) prepared a periosteum-bone complex using porcine femur, and the stiffness of the treated periosteal part was reduced to 41.6 ± 3.7 kPa. The complex exhibited the ability to enhance M2 polarization of macrophages and promote osteogenic differentiation *in vitro*, and also exhibited the ability to stimulate bone regeneration *in vivo*.

With the increasing exploration of the microscopic scale of materials, some microscopic scale parameters have also been found to be closely related to the activity of macrophages. The scaffold provides attachment sites for cells *in vivo*. In addition to affecting macrophage attachment, the roughness of the surface is also thought to affect macrophage activity. Barth et al. (2013) compared the effect of different rough titanium metal surfaces on macrophage polarization and showed that rough surfaces were more favorable for polarization of the M2 phenotype. However, Wang and his co-workers (Wang et al., 2018) reported the opposite result, noting that rough surfaces promote more polarization of the M1 phenotype. Zhang et al. (2019) indicated that only roughness within a certain range caused macrophages to exhibit an anti-inflammatory tendency and polarize toward the M2 phenotype. Abaricia et al. (2020) further pointed out the important role of Wnt signaling pathway in macrophages in response to the material surface properties, the Wnt ligand mRNA was upregulated in macrophages in a surface modification-dependent manner. In addition to surface roughness, surface wettability is similarly thought to influence macrophage activity. Hamlet et al. (2012) reported that hydrophilic modification of rough titanium surfaces reduced the expression of key proinflammatory factors. Lv et al. (2018) reconfirmed that hydrophilic modification can make macrophages exhibit anti-inflammatory and pro-healing properties, and further explained the mechanism of this phenomenon as integrin β1 and β2 affecting macrophage activity through PI3K and NF-κB pathways, respectively. Interestingly, Hotchkiss et al. (2016) compared the effects of surfaces with different wetness and roughness on macrophages, the results indicated that the polarization of M2 phenotype was better promoted on the hydrophilic and rough scaffold surface, and the wetness of the surface had a more obvious immunomodulatory effect.

As detection and preparation processes continue to evolve, scaffolds can be observed and finely processed at smaller scales, leading to the further discovery that specific topography can affect macrophages. Chen et al. (2010) reported the effect of material topography on macrophage activity in the micrometer to nanometer range. They imprinted gratings with linewidth ranging from 2 μm to 250 nm on the flat material. Under different grating conditions, macrophages exhibited different cell morphology, secretion, and adhesion states. Luu and co-workers (Luu et al., 2015) further determined that grooves with width of 400–500 nm would have the best elongation rate of macrophages, and grooves would not affect the activation of inflammatory response but would enhance the polarization of macrophages to anti-inflammatory and pro-healing phenotype. Similarly, Yang et al. (2019) compared the effects of circular patterns with different diameters (4 μm, 12 μm and 36 μm) on macrophage activity. They observed that the larger patterns promoted macrophage polarization to M2 phenotype. Interestingly, they observed that the 4 μm pattern exhibited an effect of stimulating macrophage polarization to M1 phenotype. In addition, Nouri-Goushki et al. (2021) used 3D printing technology to analyse the effects of different heights and spacing of micro columns on macrophages, and the results indicated that high enough micro columns were conducive to the polarization of M2 phenotype. However, as with many parameters, contradictory experimental findings were also reported. Vassey et al. (2020) used algorithms to generate a database of up to 2,176 micropatterns and showed that micropillars with a diameter of 5–10 μm play a predominant role in macrophage attachment, and showed that smaller and denser surface features promote M2 phenotype polarization. Furthermore, in a study comparing three different topologies (random, aligned, and the lattice), the lattice topography showed a better ability to recruit macrophages and induce angiogenesis (Jin et al., 2021). These different results may be caused by different distance settings between individual micropatterns, or it is possible that different micropatterns indirectly lead to changes in the roughness and wettability of the material surface. Considering that macrophages are in a complex 3D microenvironment *in vivo*, the 3D model can undoubtedly reflect the state of cells more formally. Jiang et al. (2016) designed a scaffold considering 3D structure, which was expanded in thickness and thus had a larger cross-section spacing than the traditional membrane structure. This scaffold showed good macrophage penetration ability and a high M2/M1 ratio in subcutaneous implantation experiments. Fang et al. (2020) further reported the relationship between 3D matrix and macrophage morphology and fusion rate in the absence of exogenous cytokines. Although 3D culture has been extensively studied in stem cells, the effects of more detailed 3D topology on macrophages still need to be investigated experimentally.

At a more microscopic molecular level, the charges and chirality of the scaffold groups are thought to affect the function of macrophages. In a previous study, the surface of titanium implants was modified with divalent cations and macrophage polarization toward the M2 phenotype was significantly enhanced (Lee et al., 2016). Mao et al. (2022) further evaluated the effect of material surface charge on macrophages and bone regeneration. Scaffold with positive surface charges significantly inhibited M1 polarization of macrophages and enhanced osteogenic differentiation of MSCs. Many substances in the body have a certain chiral selection, and chiral modification of materials has been applied to the field of bone regeneration (Xu-Kai et al., 2022). The introduction of chiral groups on the surface of materials may affect the activity of macrophages. The production of interleukine (IL) −6 and regulated on activation, normal T cell expressed and secreted (RANTES) in macrophages was better produced by the L-chirality connection of amantadine containing peptidoglycan fragments (Manček-Keber et al., 2020). Jiang et al. (2022) reported that pathology-mimetic M-nanofibers promoted macrophage M2 phenotypic polarization more than physiology-mimetic simulated P-nanofibers, significantly inhibited inflammation and promoted tissue regeneration.

**FIGURE 4:**
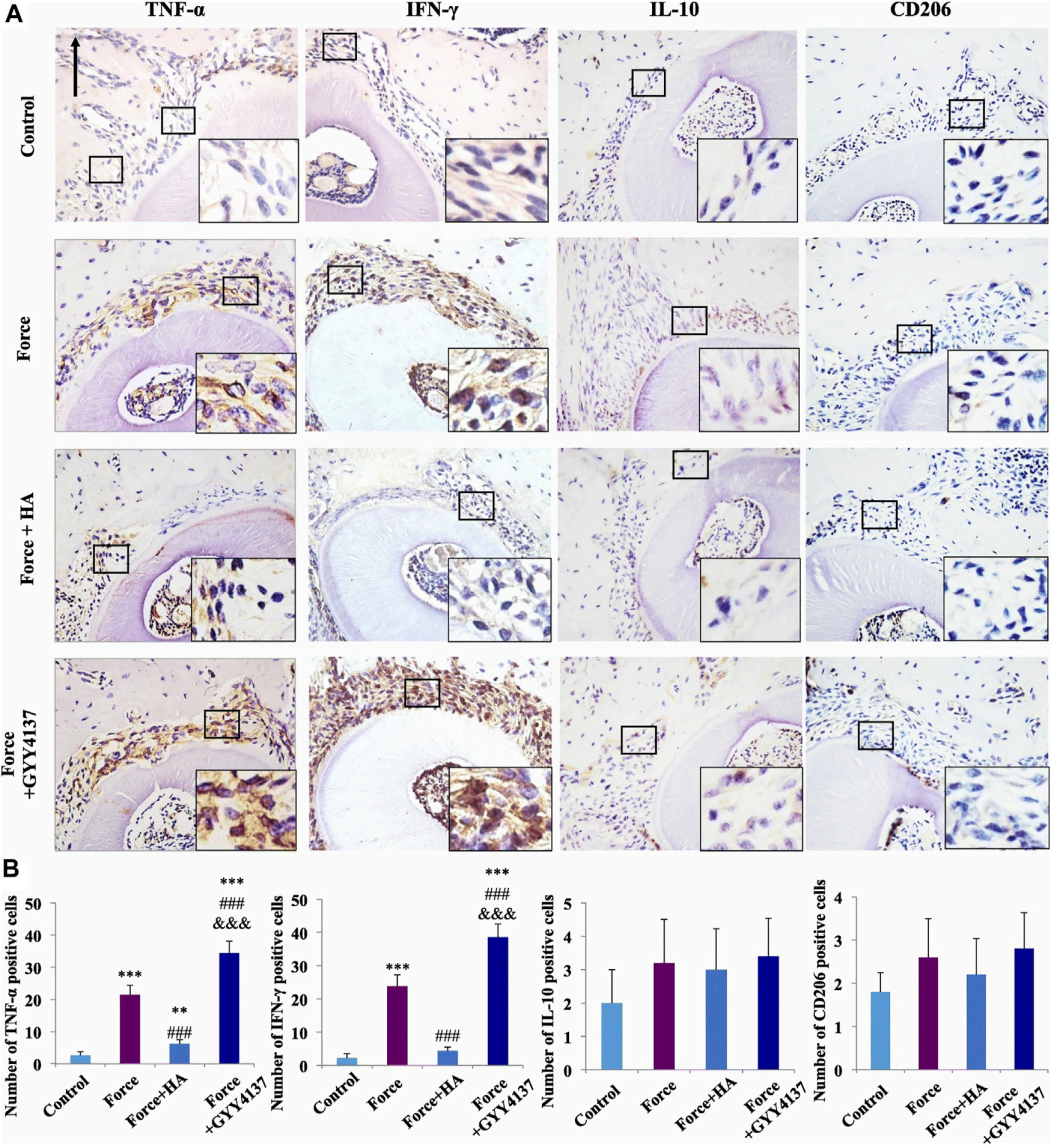
Immunohistochemical staining image of M1/M2 macrophage markers **(A)**. Semiquantification analysis of M1/M2 macrophage **(B)**. Scale bars: 100 μm **p < 0.01, ***p < 0.001 comapered with control. ^###^ *p* < 0.001 comapered with force. and ^&&&^ *p* < 0.001 comapered with force + HA. Reproduced with permission from (He et al., 2020).

In addition to the parameters embodied in the material itself, some physical stimuli also affect the activity of macrophages. Ballotta et al. (2014) reported the bidirectional effect of different cyclic strains on the polarization of macrophages in scaffold. Under 7% cyclic strain, macrophages became polarized toward M2, while under 12% cyclic strain, macrophages became polarized toward M1 phenotype. One study investigated the effect of mechanical load on macrophages and indicated that mechanical load promoted the polarization of macrophages into the M1 phenotype (He et al., 2020) (Figure 4). Stimulation through physical fields such as magnetic field, electric field, and light field has also been proved to affect the activity of macrophages, which provides new means to more fully intervene the activity of macrophages. Wosik et al. (2018) reported that external magnetic fields can rearrange the cytoskeleton, organelles, and cationic channels of macrophages and further affect the expression of molecular markers in macrophages. Similarly, Hoare and co-workers (Hoare et al., 2016) reported the regulatory effect of electric field on macrophages. Macrophages showed a tendency to move towards the anode in the electric field and reached a peak when the electric field intensity was 300 mV/mm. Interestingly, monocytes, as the precursor of macrophages, showed a tendency to move toward the cathode. In addition, the electric field also affected the phagocytosis and secretion function of macrophages. Kang et al. (2018) developed a photosensitive nanocarrier that can further regulate the polarization of macrophages by regulating the concentration of Ca^2+^ in fine macrophages through near-infrared light.

Although scientists have found many methods that can directly or indirectly interfere with macrophages and animal model techniques are becoming more sophisticated, considering the specific parameters suitable for human body and the huge differences caused by different body conditions and defect sites, a large quantity of preliminary experiments are still needed (Zhu et al., 2022b). In addition, in further clinical applications, reliable and economical production processes and standardization still need to be considered.

### 3.2 Therapeutic substance to regulate macrophage

Today, with the growing awareness that bone regeneration is an integrated process regulated by multiple factors, multifunctional scaffold systems are rapidly evolving. Their *vivo* characteristic ensures that they can intervene in multiple temporal stages of bone regeneration, and their high degree of manipulability ensures that they can meet as many environmental factors as possible that are appropriate for bone regeneration. Overall, macrophages are important targets for promoting endogenous bone regeneration, and the role of the scaffold system on macrophages is mainly reflected in the regulation of macrophage recruitment/proliferation and the M1/M2 phenotype ratio.

**FIGURE 5:**
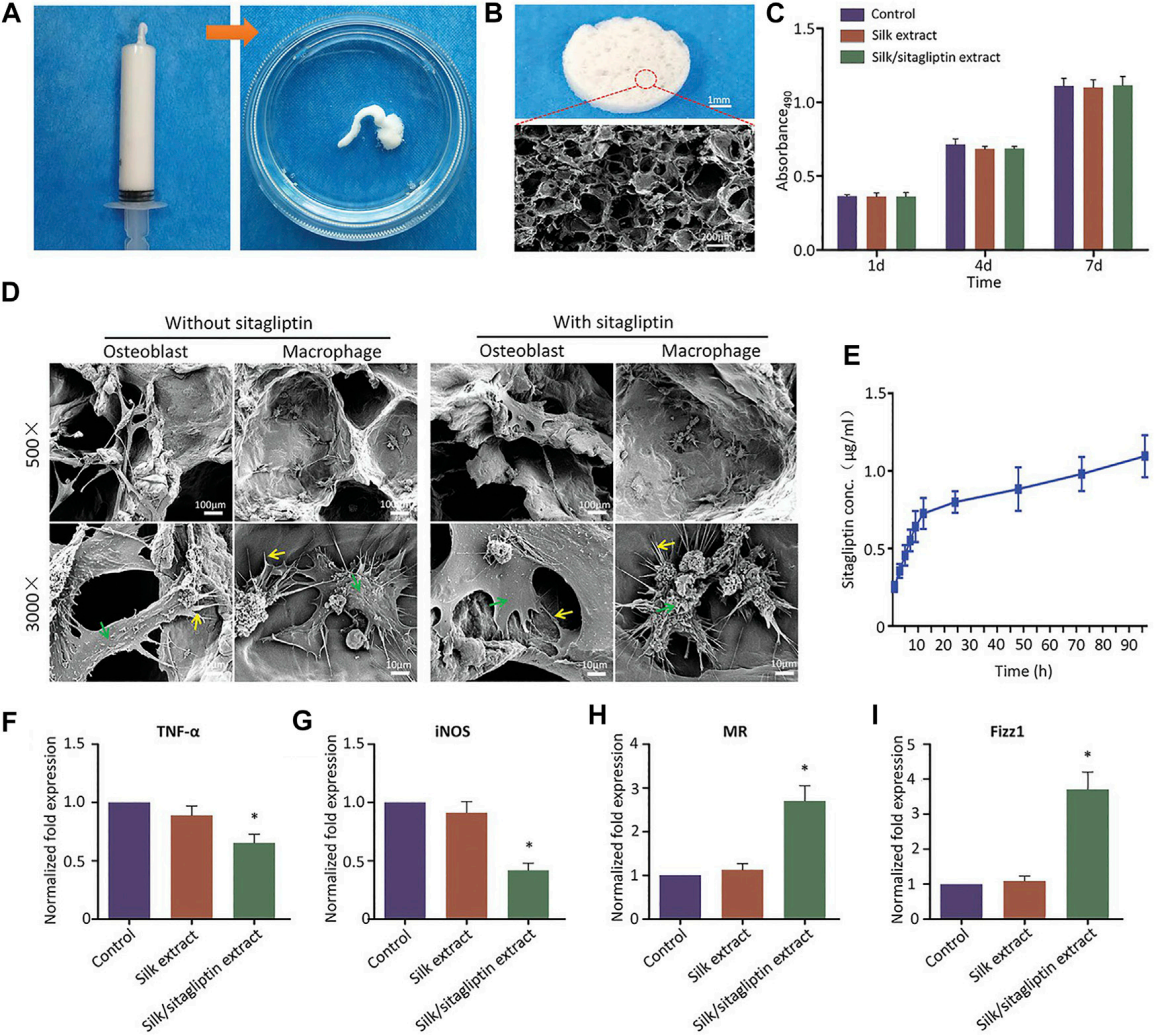
Injectable characterization of the scaffold system **(A)**. Microstructure of the scaffold under SEM **(B)**. The proliferation of mouse cells at day 1, 7, and 14 **(C)**. The attachment of macrophages or osteoblasts to scaffolds under SEM **(D)**. Drug release curve in the scaffold system **(E)**. The expression of M1-related biomarkers after 24 h **(F, G)**. The expression of M2-related markers after 24 h **(H, I)**. *p < 0.05 comapered with control group. Reproduced with permission from (Xiang et al., 2021).

An adequate number of macrophages is a prerequisite for initiating and sustaining the bone regeneration cascade. Direct delivery of allogeneic or *vitro* induced autologous macrophages may result in immune rejection or disease transmission (Chu et al., 2020; Niu et al., 2021). Therefore, there are few reports of direct macrophages promoting bone regeneration, and the mainstream methods still tend to mobilize endogenous macrophages for bone regeneration. In addition, although the excessive inflammatory response is considered to be detrimental to bone regeneration, appropriate inflammatory response and M1 phenotype macrophages in the primary stage of regeneration are also necessary for bone regeneration (Alexander et al., 2011; Edderkaoui, 2017; Schlundt et al., 2018; Pountos et al., 2019). Combining scaffold systems with chemical compounds is a common approach. Chu et al. (2019b) modified traditional collagen membranes with epigallocatechin-3-gallate to achieve better M2 phenotypic macrophage recruitment and upregulation of many growth factors and osteogenic differentiation-related factors. In their subsequent studies, they further revealed that the recruitment of M2 phenotypic macrophages may be related to the C-C chemokine receptor type 2 signaling pathway (Chu et al., 2019a). SEW2871 is a macrophage recruitment agent. Kim et al. (2014) added SEW2871 and platelet-rich plasma to gelatin hydrogel, and the hydrogel showed pro-inflammatory effects after 3 days of application, while significant anti-inflammatory effects were observed 10 days after surgery. However, in their further study, they combined SEW2871 with fibrin hydrogel scaffolds (Tanaka et al., 2019). The fibrin hydrogel alone had better anti-inflammatory effect and promoted the polarization of macrophages than gelatin hydrogel. There was no significant difference in the migration activity of macrophages whether SEW2871 was contained or not. This may be due to the rapid release and degradation of the drug in the fibrin hydrogel. A previous study reported that a sequentially controlled drug release system modulated macrophage activity (Ma et al., 2022). The system sequentially released two peptides, LL37 and WP9QY. LL37, while having an antibacterial effect, promotes local inflammation in early stage, which contributes to the polarization of M1 phenotype macrophages. The subsequent release of WP9QY has anti-inflammatory effects and promotes phenotypic polarization of M2. In addition, WP9QY also promotes calcium deposition and osteogenic differentiation. Zhang et al. (2022a) reported a method using the inflammatory microenvironment to control drug release. MnCO carried in the scaffold system would produce Fenton-like reaction with H_2_O_2_ in the inflammatory microenvironment to release Mn^2+^ and CO, thus promoting the polarization of M2-phenotype macrophages. Some traditional medicines can also be combined with scafflod systems. Xiang and co-workers (Xiang et al., 2021) prepared a silk-gel scaffold containing sitagliptin. This direct delivery method achieved local drug concentrations that were difficult to achieve through oral administration and avoided potential side effects. At the same time, the scaffold showed good anti-inflammatory effects and promoted the polarization of M2-phenotype macrophages (Figure 5).

**FIGURE 6:**
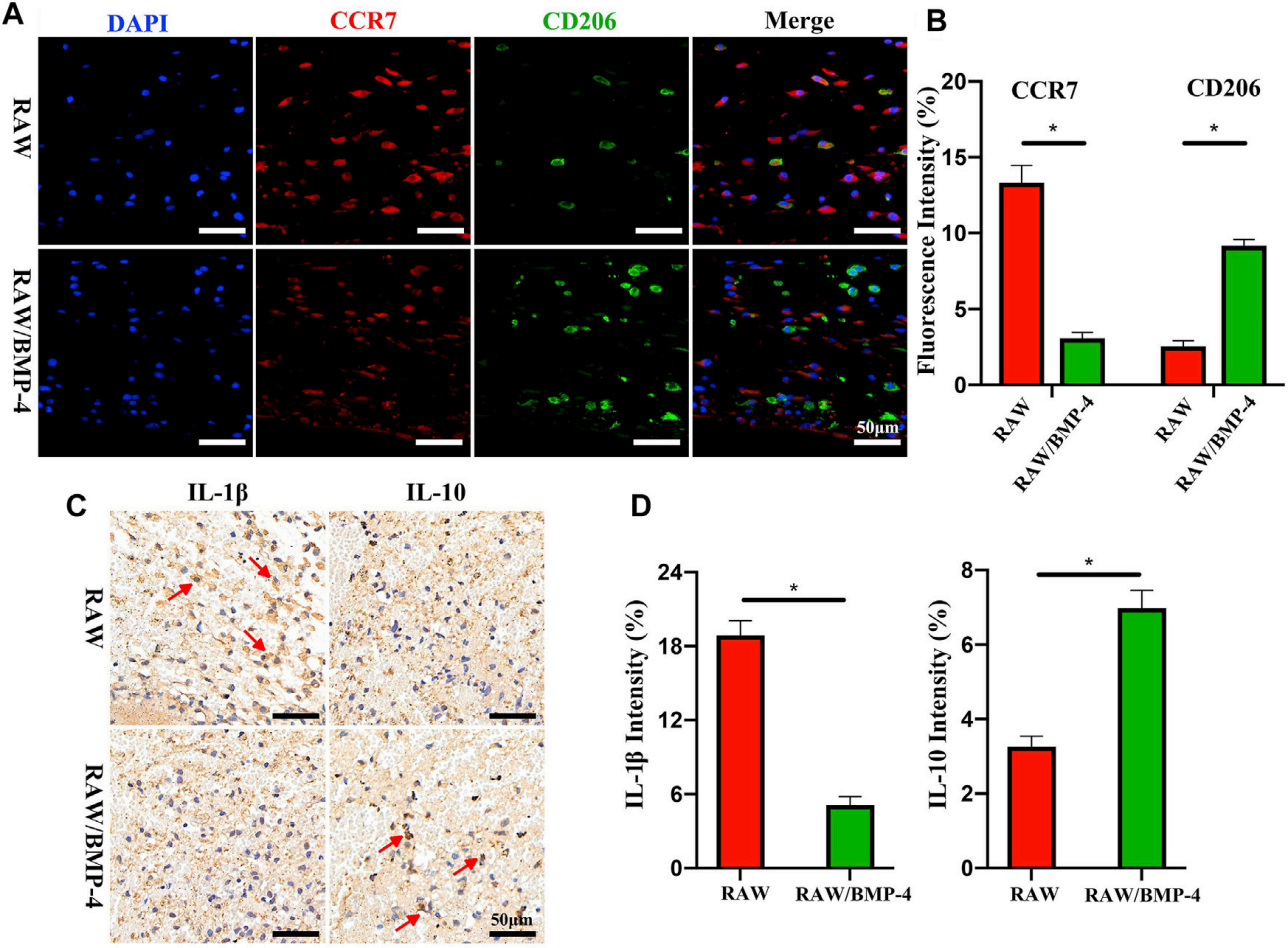
Fluorescence staining of DAPI and cells expressing CCR7 or CD206 **(A)**. Quantitative analysis of CCR7 or CD206 **(B)**. Immunohistochemical staining of IL-1β and IL-10 **(C)**. Quantitative analysis of IL-1β and IL-10 **(D)**. *p < 0.05. Reproduced with permission from (Sun et al., 2021).

Some signaling substances also show broad application prospects. Bone morphogenetic proteins (BMP) belonging to the transforming growth factor-β (TGF-β) superfamily are important substances in inducing osteogenic differentiation. As previously reported, the serum content of BMP-4 in diabetic patients decreased (Yurekli et al., 2018). Sun et al. (2021) loaded BMP-4 into mesoporous silica nanoparticles. The addition of nanoparticles enhanced the mechanical strength of the scaffold system, and the continuous release of BMP-4 promoted the polarization of the phenotype of M2 macrophages (Figure 6). Meanwhile, BMP-4 released by nanoparticles and BMP-2 secreted by M2 macrophages jointly promoted osteogenic differentiation of stem cells. Similarly, Cui et al. (2020) achieved controlled expression of BMP-4 by enabling the scaffold to carry recombinant plasmids with BMP-4 gene fragments. Interleukin-4 (IL-4) has been confirmed having an excellent regulatory effect on macrophage polarization in a large number of previous studies (Van Dyken and Locksley, 2013; Pajarinen et al., 2021). Zhao et al. (2021c) injected IL-4 subcutaneously to assist immune regulation of the scaffold system, and the results indicated that IL-4 could effectively induce M2 polarization and thus accelerate bone regeneration. Zhang et al. (2020) prepared a hydrogel bead containing IL-4, which effectively promoted the M2 phenotype polarization of macrophages and increased the expression of TGF-β1. Similarly, Ueno and co-workers (Ueno et al., 2020) prepared a macroporous gelatin-based microband scaffold containing IL-4-secreting MSCs. In the mouse bone defect model, the scaffold enhanced M2 marker expression while enhancing macrophage migration. In addition, the scaffold did not inhibit M1 marker expression. Considering the necessity of appropriate intensity of inflammation for macrophages, it is necessary to ensure the availability of a sufficient number of M1 phenotype macrophages. Chan and co-workers (Chan et al., 2015) formerly reported that the addition of low-dose recombinant human tumor necrosis factor to the fracture site in 24 h after injury enhanced bone healing in animal fracture models. Luo et al. (2021) designed an interferon (IFN)-γ/Sr-dropped bioactive glass composite scaffold. This scaffold first releases IFN-γ *in vivo* to promote the polarization of macrophages into the M1 phenotype and then releases Sr^2+^ to contribute to the polarization of the M2 phenotype. Similarly, vascular endothelial growth factor has been shown to promote macrophage recruitment in the early stages of inflammation (Hu and Olsen, 2016).

With the continuous understanding of the information transmission mechanism in bone healing, the regulatory effects of some novel signaling substances such as RNA and exosomes are also worth expecting. However, there is relatively little research on its role in regulating the function of macrophages in bone regeneration. Castaño et al. (2020) designed a collagen nano-hydroxyapatite scaffold that could deliver the antagomiR-133a. With the local release of antagomiR-133a, the number of M2 phenotype macrophages was significantly elevated, resulting in a considerable increase in bone volume in the animal bone defect model. As a means of intercellular communication, exosomes contain a variety of RNAs. Jiang et al. (2021a) developed an extracellular matrix scaffold with exosomes derived from MSCs, and the addition of exosomes effectively promoted the phenotype polarization of macrophages M2. Further, Xu et al. (2022) transfected MSCs with viral vectors containing Smurf1-shRNA to obtain engineered exosomes, which were then phagocytosed by macrophages to promote the polarization of exosomes towards M2 phenotypes using microarc titanium oxide as the delivery scaffold.

Considering the different intervention times of different scaffolds for bone regeneration and the blurred boundary of the key transition from pro-inflammatory to anti-inflammatory in bone regeneration, the regulation of M1/M2 macrophage ratio requires flexible selection.

## 4 Conclusion and outlook

With the extension of life expectancy and the improvement of health concept, tissue engineering has a broad application prospect and clinical value in the treatment of bone defects that are difficult to self-heal. Although many implants have been put into clinical application, most of them are unable to meet the requirements of promoting endogenous bone healing, especially for patients with large defect size or poor physical conditions. Poor bone healing can bring a huge burden to patients both physically and mentally. Macrophage is an important target to regulate bone regeneration, and biomaterials can provide a good medium.

In this review, we review the parameters that influence macrophages during the material design phase and the types of therapeutic agents that can be selected to further regulate macrophage activity. Although there is a wealth of excellent research emerging, the vast majority is still far from practical clinical application. To put the scaffold system into clinical application, it is necessary to master the specific parameters suitable for human tissues. However, even without considering the differences in bone parameters under different ages and nutritional conditions, the parameter differences caused by different defect sites may also be huge. Secondly, due to the blurred boundaries of each segment of bone healing, the scaffold system may face different functional requirements at different time points and periods of intervention, which not only requires careful consideration at the beginning of scaffold system design but also requires clinicians to have a correct judgment. Finally, all implants should be subjected to a rigorous safety evaluation.

In future studies, with the continuous comprehension of the mechanism of bone regeneration and the function of macrophages, more valuable targets and regulatory mechanisms will be further clarified. The rapid development of nanomaterials and the continuous improvement of manufacturing processes such as 3D printing, electrospinning, and surface modification technology make it possible to break through the bottleneck of traditional material design (Zhou et al., 2021; Wan et al., 2022). More advanced detection and imaging techniques have also enabled the mimicry of bone structures to enter a more microscopic level. In addition, the cross-integration of artificial intelligence and other fields will also greatly save the time and cost of experiments required to screen suitable parameters.
